# TriDFusion (3DF) image viewer

**DOI:** 10.1186/s40658-022-00501-y

**Published:** 2022-10-18

**Authors:** Daniel Lafontaine, C. Ross Schmidtlein, Assen Kirov, Ryan P. Reddy, Simone Krebs, Heiko Schöder, John L. Humm

**Affiliations:** 1 Departments of Medical Physics, Memorial Sloan Kettering Cancer Center, 1250 First Avenue, New York, NY 10065 USA; 2 Departments of Radiology, Memorial Sloan Kettering Cancer Center, 1250 First Avenue, New York, NY 10065 USA

**Keywords:** Molecular imaging software, DICOM viewer, Medical image manipulation

## Abstract

**Background:**

An open-source, extensible medical viewing platform is described, called the TriDFusion image viewer (3DF). The 3DF addresses many broad unmet needs in nuclear medicine research; it provides a viewer with several tools not available in commercial nuclear medicine workstations, yet invaluable for imaging in research studies.

**Results:**

The 3DF includes an image integration platform to register images from multiple imaging modalities together with delineated volumes of interest (VOIs), structures and dose distributions. It can process images from different vendors’ systems and is therefore vendor neutral. The 3DF also provides a convenient tool for performing multi-modality image analysis and fusion. The functional components currently being distributed is open-source code that includes: (1) a high quality viewer that can display axial, coronal, and sagittal tomographic images, maximum intensity projection images, structure contours, and isointensity contour lines or dose colorwash, (2) multi-image fusion allowing multiple images to be fused with VOI and dose distributions, (3) a suite of segmentation tools to edit and/or create tumor and organ VOIs, (4) dosimetry tools for several radioisotopes, (5) clinical tools for correcting acquisition errors, including patient orientation, and (6) the ability to save the resultant image and VOI as DICOM files or to export the numerical results as comma separated values files. Because the code is written in MATLAB™, it is highly readable and is easier for the coder to make changes compared to languages such as C or C++. In what follows, we describe the content of the new TriDFusion (3DF) image viewer software platform using examples of a number of clinical research workflows. Such examples vary in complexity but illustrate the main attributes of the software.

**Conclusions:**

In summary, 3DF provides a powerful, convenient, easy-to-use suite of open-source imaging research tools for the nuclear medicine community that allows physicians, medical physicists, and academic researchers to display, manipulate, and analyze images.

## Background

Nuclear medicine is a specialty that requires advanced image processing and display software to allow physicians and scientists to quantitatively visualize molecular images and therapy agents to monitor disease progression, to determine treatment response, or to perform dosimetry for radionuclide therapy. To meet these objectives, the software should be both integrated into the image generation workflow (image acquisition, reconstruction, processing, and viewing) and able to facilitate the integration of diverse molecular imaging studies with anatomical imaging modalities such as computed tomography (CT) or magnetic resonance imaging (MRI). Several vendors have developed software with such capabilities [1–6], but in general these do not have the flexibility of 3DF that allow physicians/scientists to customize the code to meet their research needs. An example where a vendor solution may not exist is if a technologist inadvertently selects the wrong patient orientation for a scan acquisition, there is no convenient tool to correct the header orientation so that the image series can be subsequently reopened in the appropriate orientation. Also, because a vendor’s solutions are often dedicated to its own products, these problems are often complicated in hospitals with a mixed vendor environment, which is typical for larger institutions. Several research workflows that employ new processing or assessment tools often have limited interfaces with vendor software. In this work, an open-source software platform that can address such an unmet need is described.

The applications of imaging system vendors [7–12] and vendor-neutral software companies’ [1, 2, 13, 14] provide several tools required in a nuclear medicine clinical environment as well as performance display tools to facilitate image reading. Typical features offered by vendor-neutral software include: (1) image analysis to support the diagnosis of a wide variety of conditions (e.g., kidney, cardiac, lung and gallbladder function, and gastric emptying) [2, 15, 16] as well as therapeutic applications (e.g., lung shunt fraction, thyroid and salivary gland uptake) [2, 15, 16], (2) segmentation of organs and disease [1, 2, 5, 17], (3) image registration and fusion [1, 2, 5, 6], (4) dynamic image display [1, 2], (5) single-photon emission computed tomography (SPECT) image reconstruction [2, 18], (6) organ and voxel-based dosimetry [12, 19, 20], and (7) kinetic modeling [21]. For a commercial vendor, the decision of adding new features or modifying existing ones is a balance between the benefit to the company and the associated cost of implementing the user request. To address this problem, several vendors offer software development kits (SDKs). However, their use can be complicated because vendor source code is proprietary, lacks transparency, and the SDKs generally require substantial programming experience. Also, the SDKs often do not provide a flexible means of integrating the resultant images/data back into the application or can introduce complicated, inefficient workflows. Finally, the dissemination of the resulting tools is dependent on the vendor maintaining a software repository to organize and unify the resultant tools.

The research community has responded to the difficulties faced by the vendors by introducing several free applications that provide platforms on which to develop and test their tools [22–25]. In particular, ImageJ and 3D Slicer have become popular in the research community by offering image display tools that allow users to define plugin modules to perform specific tasks [22, 23]. Usually, these plugins are designed by users to perform highly specific tasks available for download. Complex tasks, usually performed using macros, may require searching for multiple plugins, often from different sources, to be used together which adds complexity and increases the potential for compatibility problems.

In this publication, the open-source imaging toolbox TriDFusion image viewer (3DF) is introduced to address a number of unmet needs of the research nuclear medicine communities. The 3DF application first and foremost provides a vendor-independent open-source viewing platform that allows users to display and export both images and data generated using the users own analysis tools. Because this software is open source, the viewing platform can be utilized to display and export the results of any customized workflow. Tools built by researchers can be uploaded to the software repository and shared within the research community, who will collectively contribute to their maintenance and compatibility. To illustrate the power and utility of the 3DF application, clinical research examples of the basic and special workflows available on the platform are provided.

## Methods

### A general description of the TriDFusion (3DF) image viewer

The TriDFusion image viewer (3DF) is a nuclear medicine research tool built on an open-source MATLAB™ platform. To improve its utility, it has several general features that are designed to mimic commercial applications with the addition of multiple powerful and versatile image editing, manipulation, and display tools. Such features include generic operations such as: image display in two-dimensional (2D) and three-dimensional (3D), registration, fusion, segmentation, and arithmetic. The 3DF software repository can be accessed from GitHub (3DF-download). In addition, it is compatible with several software platforms, including DICOM Database Browser (DBB) (DDB-download) and the Computational Environment for Radiological Research (CERR) (CERR-download) [25]. The 3DF software can be compiled with DDB software, which is an application for browsing DICOM data and launching applications such as 3DF, CERR or other software.

**Fig. 1:**
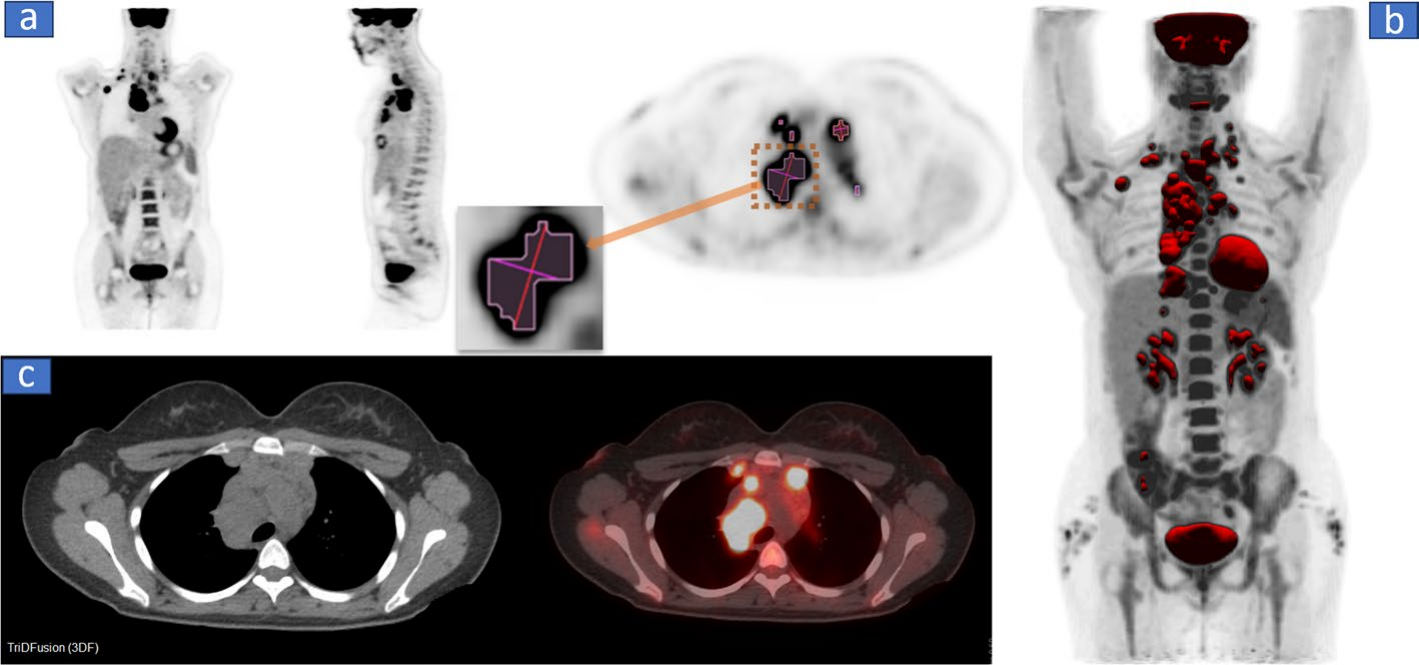
**a** The standard coronal, sagittal, and axial positron emission tomography (PET) displays in grayscale with segmented volumes of interest. The maximum intensity projection displayed in **b** is fused with the isosurfaces. Note the RESIST distance tool shown in the exploded view. **c** The transaxial computed tomography (CT) and PET/CT fusion images

Figure 1 shows a montage displays of a positron emission tomography/computed tomography (PET/CT) image. Figure 1a shows the standard coronal, sagittal, and axial display. The 3DF has tools to segment the image manually or automatically. A unique capability of the software is to fuse the isosurface contours with a maximum intensity projection (MIP) display as shown in Fig. 1b. The software can also display more traditional views, including transaxial CT and PET/CT fusion images as shown in Fig. 1c. TriDFusion can view data sets consisting of 2D, 3D, and four-dimensional (4D) DICOM images and is compatible with several imaging modalities and file formats, as shown in Table 1.

**Table 1:** TriDFusion image viewer modality and file format compatibility

Medical imaging modalities	Import/export file formats
Positron Emission Tomography PET-CT (PT)	DICOM using custom/vendor dictionaries
Gamma Camera, Nuclear Medicine (NM)	Raw data from nuclear imaging devices
Computed Tomography (CT)	DICOM-RT structure (VOI)
Digital Radiography (CR, DX)	CERR planC, dose volumes and constraints
Digital Angiography (XA)	Comma separated values (.csv)
Magnetic Resonance (MR)	Standard triangle language (.stl)
Secondary Pictures and Scanned Images (SC)	Bitmap (.bmp)
Mammography (MG)	
Ultrasonography (US)	

CERR, Computational Environment for Radiological Research; VOI, volume of interest

### TriDFusion (3DF) image viewer general features

Nuclear medicine clinical research requires a diverse range of software tools to manipulate, reformat, and process images. This toolkit provides the capability to test and validate different techniques, as well as correct images with acquisition errors.

### Overview of general features

**Fig. 2:**
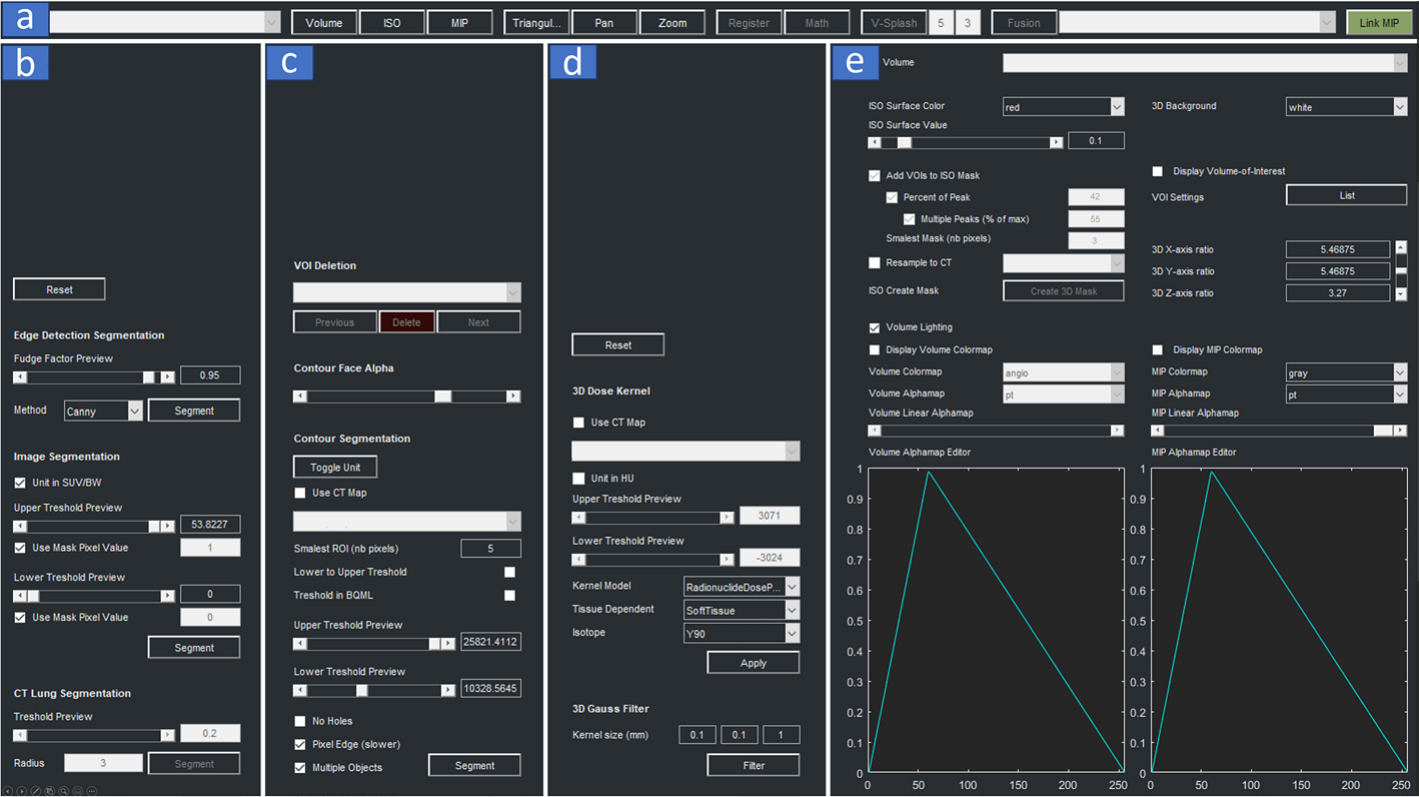
The main ribbon and pull-down menu panels. Ribbon **a** on top. Below the ribbon are the various tools from left to right: **b** image editing, **c** contour segmentation, **d** kernel, and **e** the three-dimensional rendering. Selected features from these panels are shown in Table 2 and discussed in “Select features from these p” section

The default panels of the 3DF are displayed in Fig. 2. The individual features are selected via buttons and pull-down menus from the main ribbon. Some of the imaging toolbox features are listed in Table 2.

**Table 2:** The panel column indicates the ribbon or pull-down menu shown in Fig. 2

Item	Panel	Description
1	Figure 2a: toolbar ribbon	Display of images in 2D and 3D view
2		Image fusion between modalities
3		Image registration
4		Image resampling, change field of view
5		Edit/correct image orientation
6		Image arithmetic and filtering
7	Figure 2b: image panel	Image object thresholding for image segmentation
8		Lung segmentation
9		Edge detection
10		Image mask
11	Figure 2c: contour panel	Contour generation based on SUV, count or HU based thresholding

Selected features from these panels are discussed in “Main features of TriDFusion (3DF)” section. SUV, standard uptake value; 2D, two-dimensional; 3D, three-dimensional

#### Main features of TriDFusion (3DF)

1. *Image viewer* Multi-dimensional viewing capabilities of two-, three-, or four-dimensional images can be displayed with the ability to also triangulate within the fused image display. Furthermore, the 3D fusion view uses 3 different techniques that can be exported as a gif file. A volume rendering can be fused with the isosurface and MIP. The intent is to have the MIP, the isosurface, and the volume rendering fused together to help locate the regions of focal uptake from PET or SPECT medical images. The order of activation of each technique is important because it dictates the emphasis of each feature in the resultant image. For example, if the isosurface is activated last, that layer will appear on top of the other layer(s) MIP and/or volume rendering. Opacity can be manually adjusted to avoid obscuring the lower layers. The color and alpha maps of the MIP and volume rendering are fully adjustable and set independently to allow for isolating specific layers for analysis. The isosurface (level-set) value is adjustable. Additionally, all three 3D techniques are computed from a group of 3D DICOM images and acquired in the axial, sagittal, or coronal plane by PET, SPECT, CT, MRI, or micro-scanner.
2. *Image fusion* The ability to fuse and display multiple imaging modalities or tracers in 2D or 3D allows multiple images to be easily and rapidly compared with one another. Multiple series can be fused to a reference series. The matrix from the reference image is selected by the user (can be CT, MRI, or NM) and will be used to resample the secondary images series. When fusing more than two series together, the color, window and level, and relative intensities can be adjusted independently of one another. For example, PET/CT and MR studies can be fused as a single image where each series’ windows and levels can be independently altered to help compare metabolic uptake with anatomical features. As another example, multiple PET studies, possibly with different tracers, can be fused to a reference CT, where the CT maintains grayscale, and each PET study is assigned a particular color scale and intensity. In each case, the underlying CT Hounsfield units or standard uptake value (SUV) are displayed in the fused series. The values depend upon the type of interpolation selected. The user can select between nearest, linear, cubic, bilinear, or bicubic interpolation schema. Interpolation using nearest neighbor will preserve the original voxel value of the fused resampled image. The fused series can be moved or rotated manually while preserving its relationship to the reference image series. The resultant fused images can also be exported as a new DICOM series.
3. *Image registration* The ability to register multiple image series with a selected reference has multiple uses. For example, the display of flipbooks, in which a series of brain studies MRI, PET, SPECT, and CT are co-registered and resampled in the selected frame of reference. The axial field of view displaying a tumor can now be shown as a function of scan date in a versatile dynamic presentation. Furthermore, the lesion can be segmented so that the tumor volume can be assessed from baseline through treatment and treatment follow-up. In addition, following image registration the slices of the secondary image series are interpolated to match those of the reference image. Also, a splash display in axial, coronal, or sagittal formats can be presented using a gated display format to allow the physicians to readily visualize tumor changes over the patient’s scan history. The “gated” display can be exported as a.gif file.
4. *Image resampling* The default image display of 3DF is the original acquired format. Resampling one modality to another will transform the matrix dimensions of the secondary images (e.g., additional images, structures, and regions of interest [ROI]) to that of the reference image format. The measurement and values will then be computed using the resampled image. The resample values can be computed by different interpolation methods (nearest, linear, cubic, bilinear, and bicubic). ROIs within the up (or down)-sampled images will be resampled to the reference image values, e.g., CT.
5. *Image reorientation* Images can be resampled from any sectioning plane (axial, coronal, or sagittal) to any desired oblique plane. For instance, patient images acquired using incorrect superior/inferior and/or left/right orientations can be reoriented to their correct position.
6. *Image arithmetic and post-filtering* Both arithmetic and post-filtering operations can be performed among any image sets. For example, given image (A) and image (B), you can perform any mathematical operation (supported by MATLAB™, including scripts) between the two. A relevant application would include scatter correction in a SPECT image. Post-filtering can be performed using 2D or 3D Gaussian filters as well as a user-defined spatially variant kernel. This functionality could be used to help harmonize the images acquired from PET and SPECT scanners and/or reconstructions with different resolution properties.
7. *General segmentation* The 3DF supports versatile thresholding capability, e.g., min to value, value to max or between two values. Thresholding can be performed over the entire image, inside or outside of constrained regions, which serve as exclusion zones. Selected voxels can be replaced by custom values. The results can be presented with or without inclusion of data from the exclusion zones. Examples include tumor within a bounding box, blood volume with a VOI on a contrast CT or MR, skeleton, etc. The processed images can be exported as a new DICOM series.
8. *Lung segmentation* A separate dedicated tool exists to segment right and left lung volumes thereby allowing the user to perform the operations described under general segmentation on the contents of the entire lung or segmented lung sub-volumes. The resultant image can be used to generate isocontours, which can be exported as a mesh grid (.stl), a new DICOM series, or ROIs generated and exported as RT structure sets.
9. *Edge detection* Edge detection using the Sobel, Prewitt, Canny, or Approx-Canny can be applied to a series, and the resultant edge contour can be used to assist and/or verify the quality of the fusion between two modalities. The edge images can be displayed in either 2D or 3D and exported as DICOM masks.
10. *Image mask* It is possible to mask voxel values that the user does not wish to be included in the analysis. These include structures external to the patient such as the table, prostheses, pacemakers, or the contents of the bladder can be assigned to a custom value. This functionality provides a way to change voxel values prior to generating a model for 3D printing such as a specific bone structure or a radiotherapy positioning mold.
11. *Total tumor burden determination* There is a common need to determine the total tumor burden within a patient. Objects can be aggregated as a single VOI. Such aggregation allows statistical analysis to be performed across an entire volume, representing the total tumor burden, and can also be used to segment the total mass, e.g., skeleton, prostheses, etc. The software allows contours to be generated within the mask, e.g., bronchi within a lung mask. The contours can be exported as a RT structure set or DICOM mask.

### Workflow examples of viewer features

In this section, we provide several useful clinical research examples to demonstrate the utility of the 3DF image viewer.

### 3D visualization with volume rendering

**Fig. 3:**
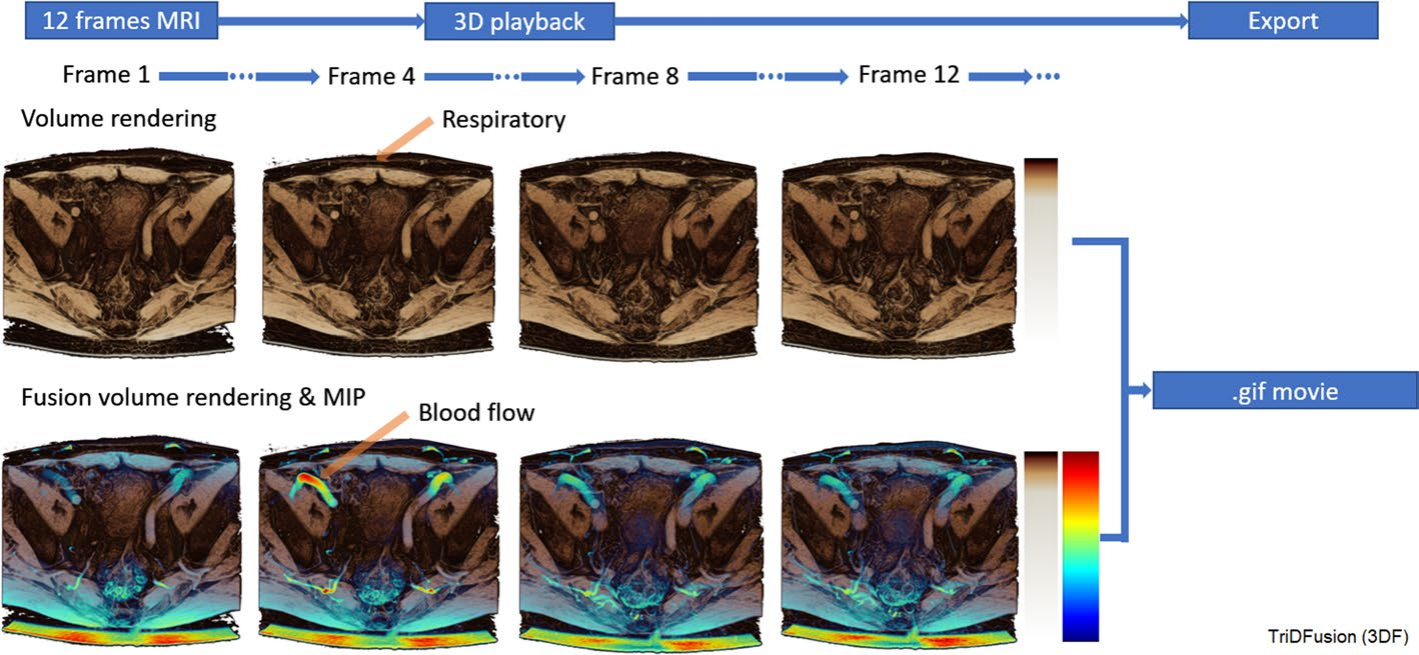
A three-dimensional volume rendered view of a four-dimensional magnetic resonance imaging sequence of the soft tissue in the pelvis (row 1) fused with a maximum intensity projection (row 2) to simultaneously highlight respiratory motion and blood flow

Figure 3 shows a 3D volume rendered view of a dynamic MRI sequence of the soft tissue in the pelvis. These data can be shown in cine mode (but are shown as splash display for this publication) to present a retrospective time display of a moving organ. Also shown are the same images fused with a MIP to highlight blood flow. The ability to select different color and alpha maps for each technique enables the display of two salient features of the data in the same image.

### Image fusion

**Fig. 4:**
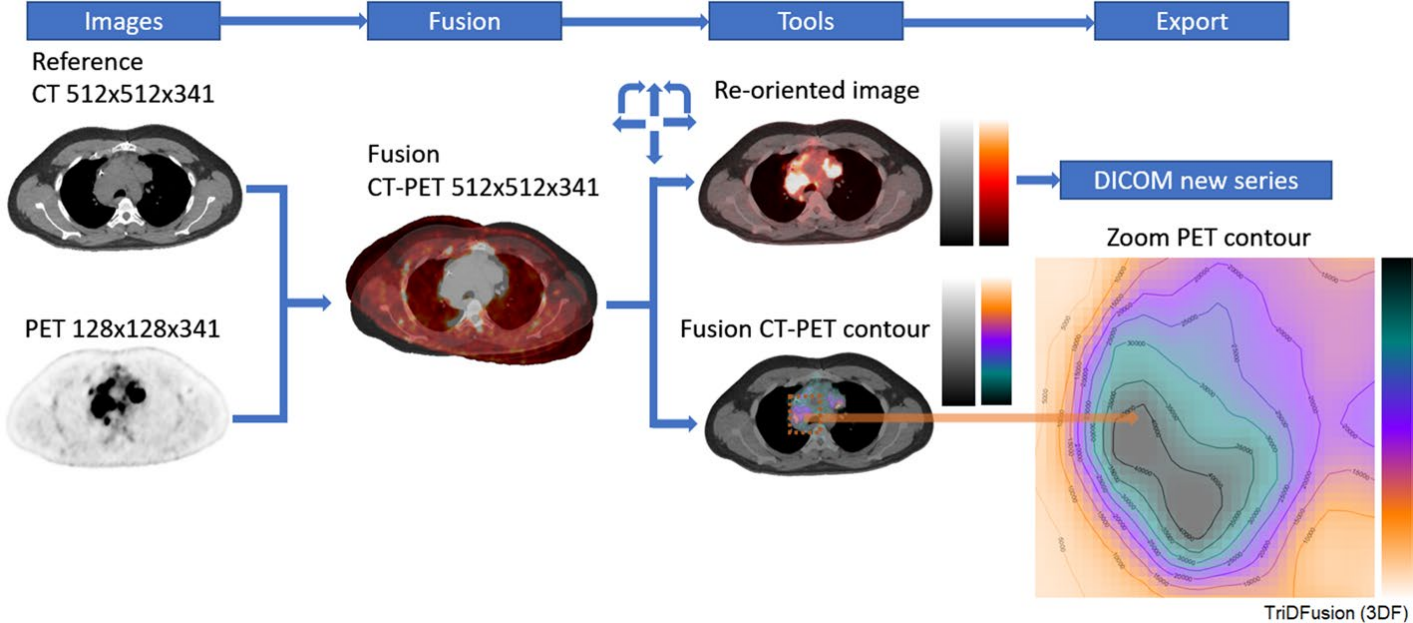
Image fusion workflow using computed tomography (CT) and positron emission tomography (PET) images

The image fusion tool allows image sets to be initially resampled to match a reference image series in full 3D. The resultant subordinate image series can be manually reoriented as needed. Basic image processing can be performed on any of the subordinate image series in the fusion space. Figure 4 shows an external PET that was manually registered to the reference CT. Isocontour maps, representing radiotracer uptake in the 3D fusion space, are generated from the PET image. Such maps can be fused with the reference or any other co-registered subordinate image series. There is built-in versatility to change the color setting and the values. Image fusion can be performed on any modality, including dynamic acquisition series, planar multi-window images, or multiple whole body gamma camera images.

### Image registration, reformatting, and flipbook display

**Fig. 5:**
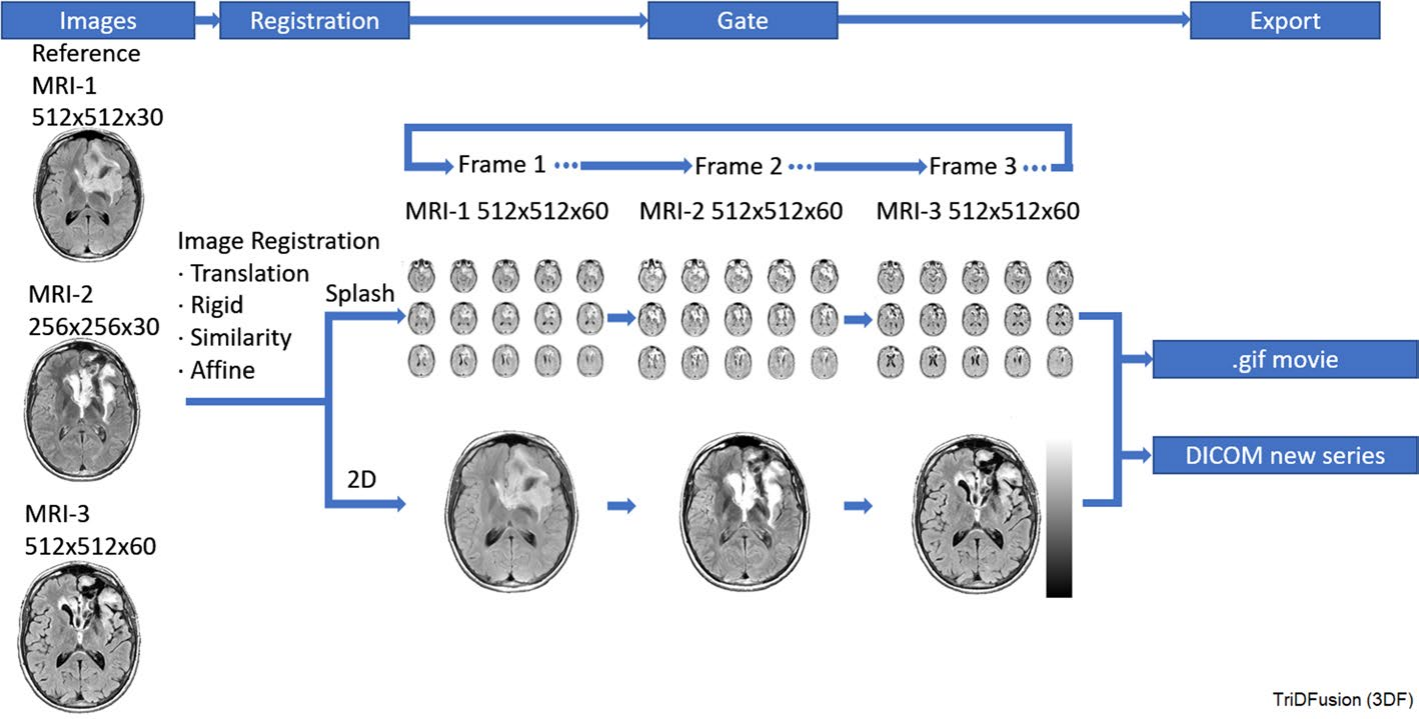
Image registration workflow to create a flipbook using three separate magnetic resonance images (MRIs)

Multi-modality images from several time points can be co-registered to a reference image providing a useful display to dynamically visualize disease progression or response during or following treatment. Registration is performed using rigid, similarity or affine transformations with linear, cubic, bilinear, bicubic or nearest neighbor interpolation. The registered images can also be displayed as a v-splash series and played in time. Figure 5 shows 3 separate MRI images that are registered and reformatted to a reference image format series to create a flipbook. The resultant dynamic image sequence can be exported as a.gif, and the registered series can be exported as a new DICOM series.

### Image resampling

**Fig. 6:**
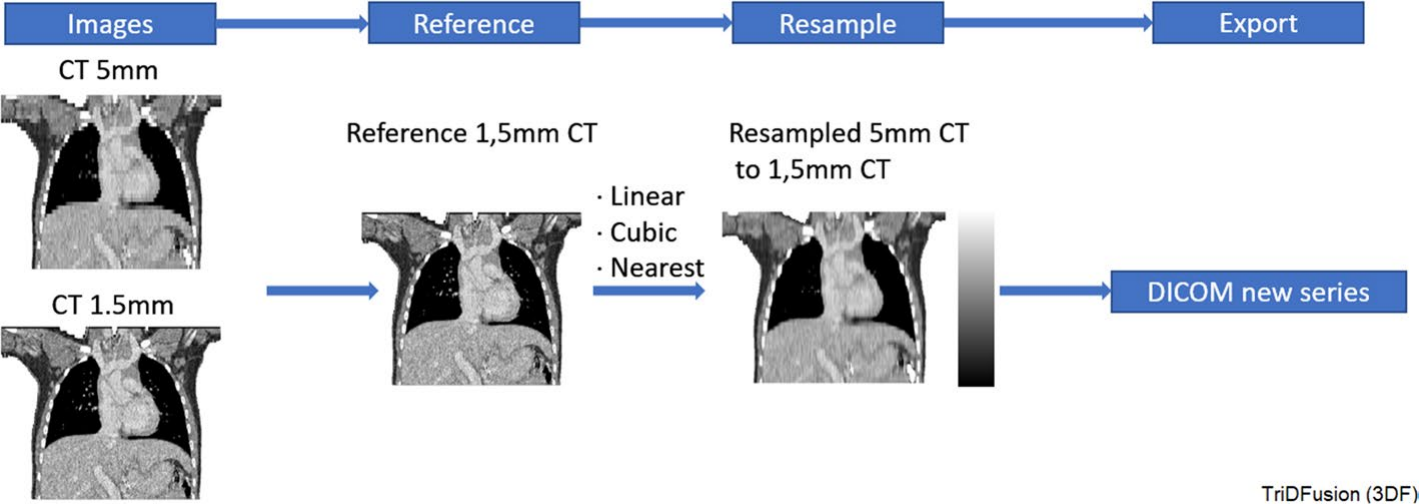
Image resampling workflow using two computed tomography (CT) images

The 3DF supports resampling of a modality, such as a PET image, to the same matrix format as a co-registered CT dimension or vice versa. When up-or-down-sampling occurs the intermediate voxel values are interpolated. The measurement and values will then be computed from the resampled image. The kernel is resampled to the voxel size of the image by using one of the following interpolation schema nearest, linear, cubic, bilinear or bicubic. For example, if the resolution of a convolution kernel is in 1.5 mm intervals and the image data have a 5 mm voxel size, then the image can be up-sampled to improve the accuracy of the image convolution. Figure 6 shows a CT with thicker (5 mm) slices up-sampled to match a reference CT with thinner (1.5 mm) slices.

### Image reorientation

**Fig. 7:**
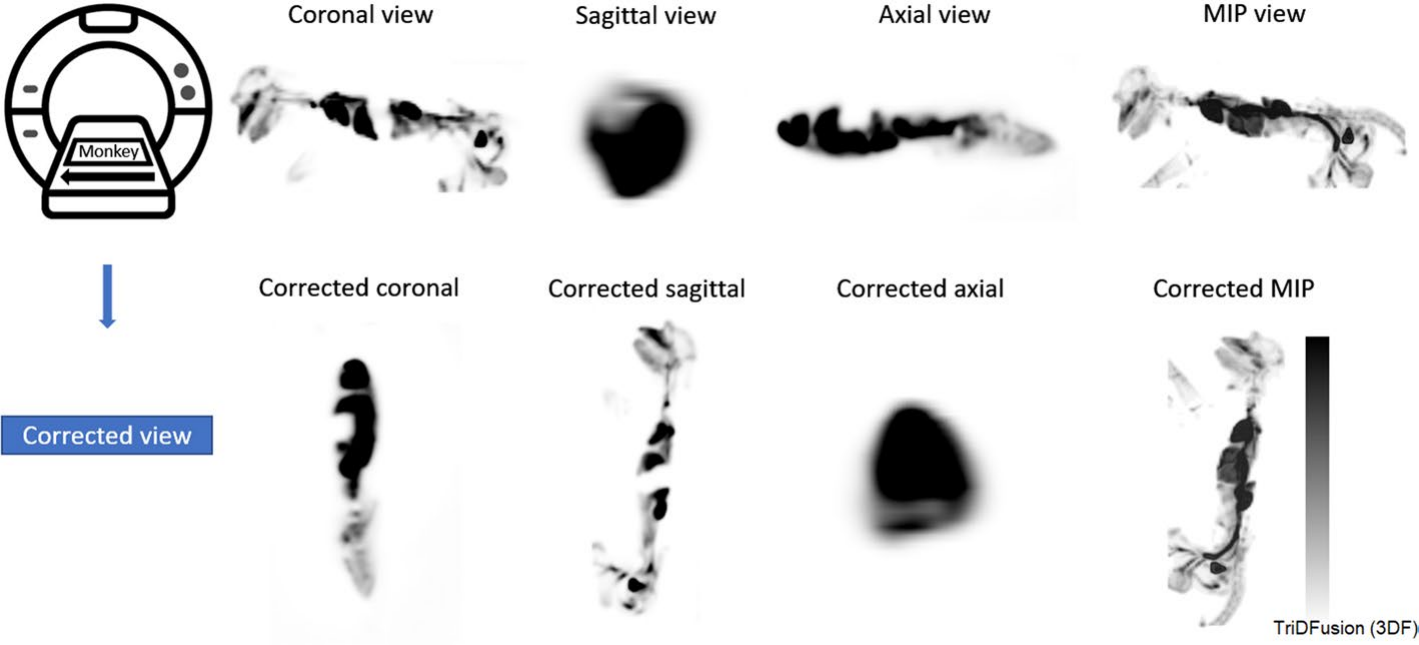
Image orientation correction workflow using a positron emission tomography image of a monkey acquired in the lateral position. Top row: reference images before reorientation using TriDFusion (3DF) image viewer; the listed orientation is incorrect. Bottom row: reference images after reorientation; the listed orientation is correct. MIP, maximum intensity projection

This tool allows the reorientation of an image volume to correct discrepancies between the patient acquisition alignment and the patient’s physical orientation. This can occur as an accidental misalignment on the acquisition console or intentionally when a patient is unable to lie supine or prone, such as during an interventional radiology procedure. Figure 7 shows a monkey that was placed laterally in the scanner to image the entire animal within a PET single field of view. Because the scanner console does not support the monkey’s orientation, the images shown between the top and bottom rows are mismatched. The resultant images can be saved as a new DICOM series.

### Image arithmetic and post-filtering

**Fig. 8:**
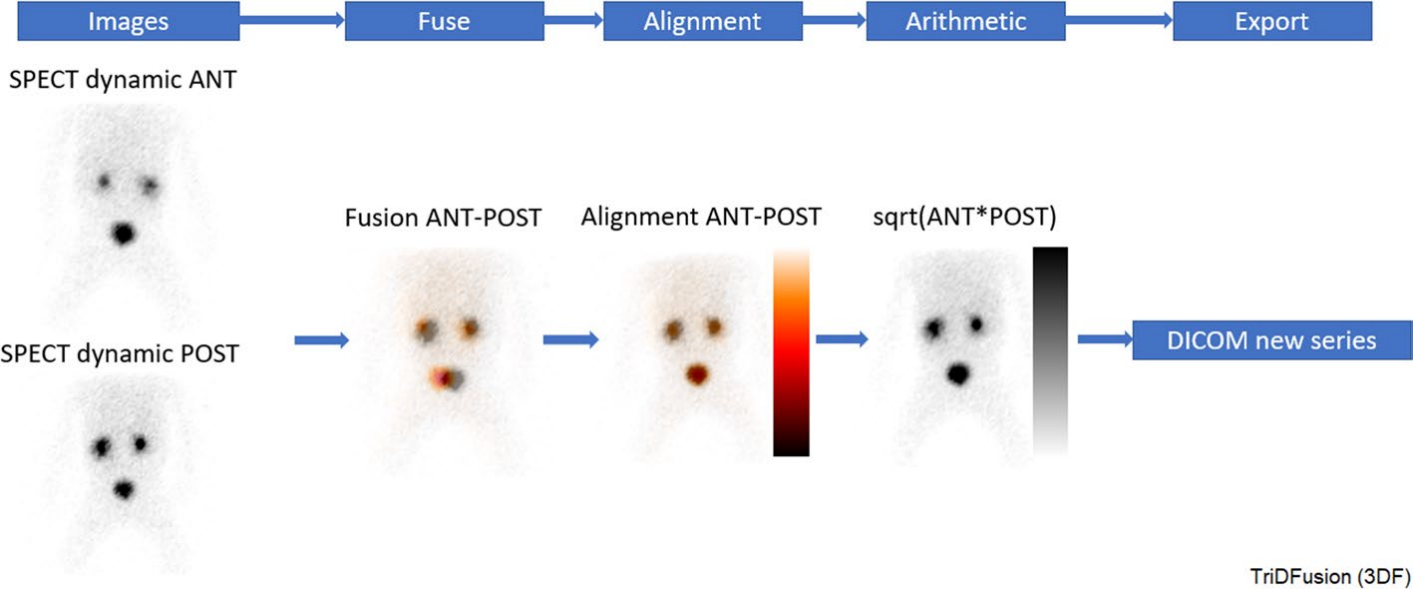
Workflow to create a geometric mean image using two single-photon emission computed tomography (SPECT) images. ANT, anterior; POST, posterior

Figure 8 shows the workflow to create a geometric mean image on a dynamic image series using the image arithmetic tool. The nuclear medicine ANT and POST frames are displayed, the ANT frames are then flipped and co-registered (as necessary to correct for any center of rotation displacement), and a geometric mean image is created.

### Blood pool segmentation

**Fig. 9:**
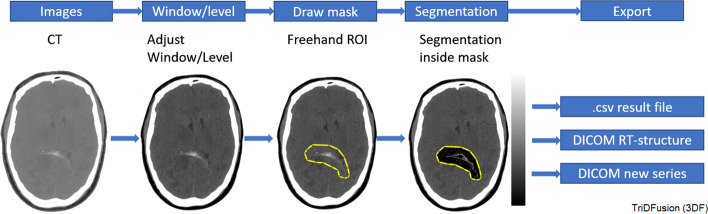
The workflow to isolate blood from the surrounding tissue. The sequential steps for blood pool segmentation are shown from left to right. The yellow border indicates the constraint, and the isolated voxels are depicted on the right. CT, computed tomography; ROI, region of interest

Blood pool estimation can be performed on a window/level adjusted contrast CT within a user-defined VOI (freehand or automated). Figure 9 shows the image segmentation operation confined to isolate the blood pool within the VOI mask. The resultant nonzero voxels can then be counted to estimate the blood pool volume.

### Lung segmentation

**Fig. 10:**
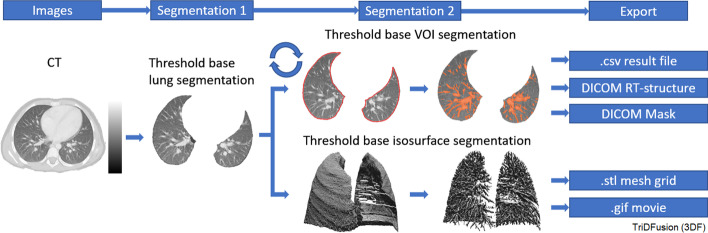
Lung segmentation workflow of a computed tomography (CT) image segmented using the lung segmentation tool. The workflow illustrates how the resultant image (bronchi) is then segmented using the contour tool or the three-dimensional isosurface tool and exported. VOI, volume of interest

Figure 10 shows segmented lung lesions from CT and a 3D isosurface created by selecting a threshold to include the entire lung bronchi. The delineation of the lung fissures can be used to evaluate pulmonary function or lesion classification and burden within the individual lung lobes. The resultant VOIs can be copied onto co-registered modalities, e.g., SPECT or PET. These isosurface volumes can be saved as.stl mesh grid for 3D printing or as a.gif file.

### Edge detection

**Fig. 11:**
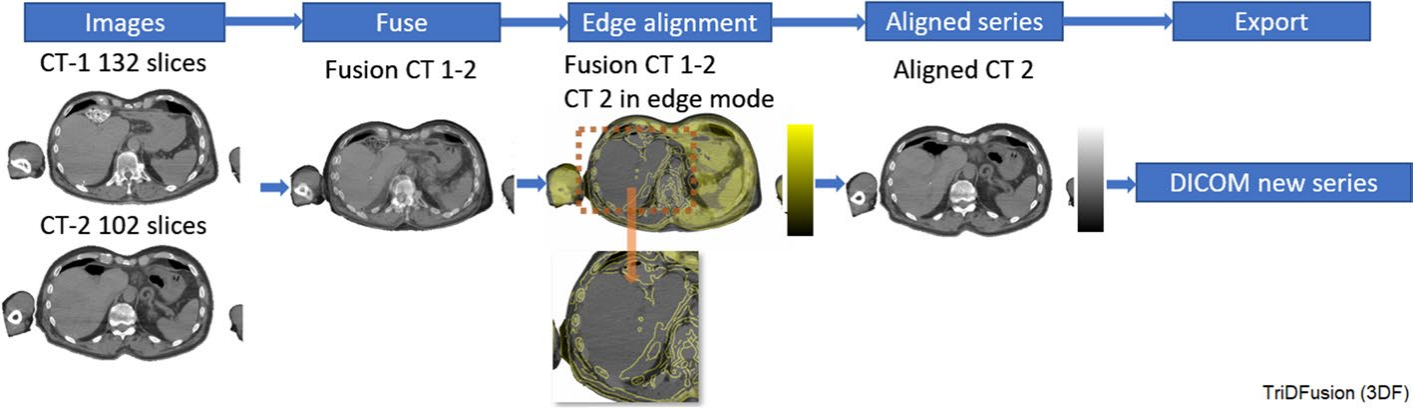
Image edge detection workflow for fusion of misaligned image using a constraint (orange box) of computed tomography (CT)-1 fused with CT-2 to resolve the misalignment

Edge detection is used to assist with the alignment of a second CT to a reference CT via the fusion tool. Figure 11 shows a workflow where edge detection helped resolve a misalignment between images of the liver, a consequence of different phases of the breathing cycle. Both the color and edge detection methods are customizable (Sobel, Prewitt Canny, and Approx-Canny).

### Confounding structure removal

**Fig. 12:**
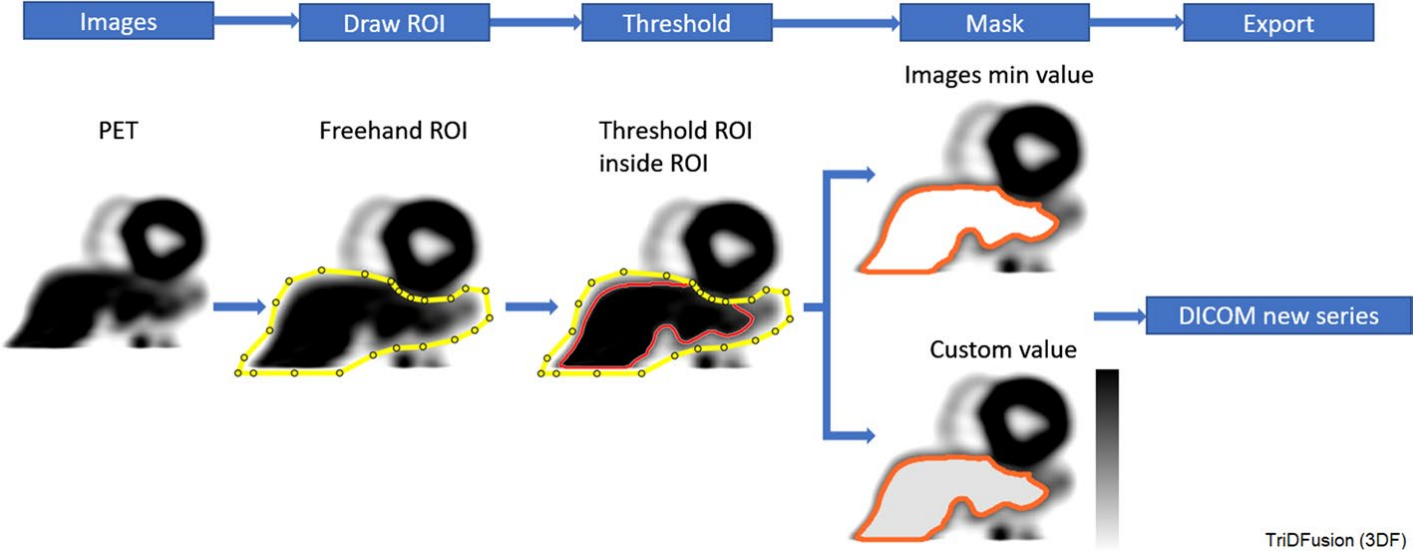
The workflow to remove confounding structures. The example depicts a heart/liver mask using positron emission tomography (PET) images. The yellow border indicates a constraint whereas the orange border indicates a contour created by a threshold. The gray area indicates the mask of the contour. ROI, region of interest

A common problem in image analysis from nuclear medicine studies arises when an organ abuts the structure of interest. Figure 12 shows a signal from the liver can confound software designed to assess cardiac blood flow. A manual bounding region can be rapidly drawn to constrain the auto-segmentation of the liver. Once performed, the liver voxel values can be removed or assigned a custom value.

### Multi-threshold segmentation

**Fig. 13:**
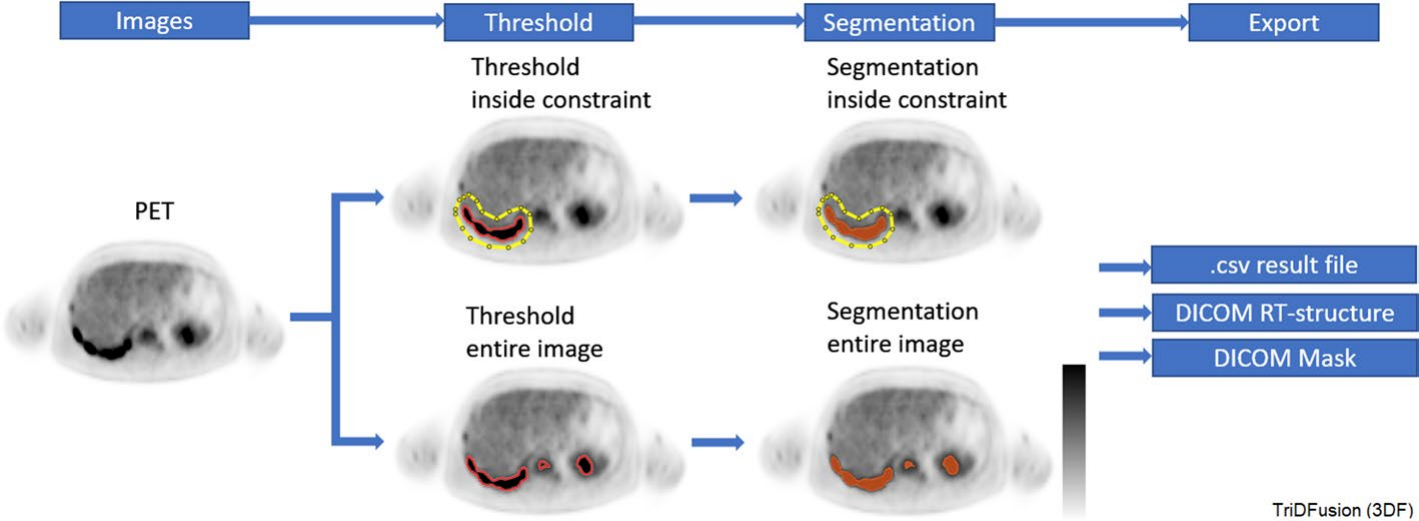
Workflow for multi-threshold segmentation using the contour tool on a positron emission tomography (PET) image. The orange outline indicates a preview of the contour. The filled region (orange) is the resultant contour from a constraint (yellow) or the entire image

This tool allows user-defined constraint to generate contours from thresholds. The workflow in Fig. 13 shows how individual metastatic lesions could be performed based on a percentage of the local maximum constraint.

### TriDFusion (3DF) image viewer special features

### Overview of special features

The 3DF image viewer also supports workflows that utilize multiple panels or requires several steps. These more complicated features are listed in Table 3 and described in the sections below.

**Table 3:** TriDFusion image viewer special features

Item	Panel(s)	Description
1	Figure 2a	2D multi-fusion
2	Figure 2d	Image convolution w/user-defined kernel (entire image or Kernel Panel under a mask)
3	Figure 2e	3D isosurface mask of target tumors or organs in DICOM 3D Panel image format
4		3D printing, isosurface base model export in .stl file format

2D, two-dimensional; 3D, three-dimensional

1. *Multi-fusion* This tool allows multiple registered image series to be displayed fused with a reference. Each fusion can be displayed with different color and alpha maps, providing a mechanism to highlight the salient findings of each image series.
2. *Image convolution* This tool allows image convolution with a user-defined custom 3D kernel. One use of this tool is to help harmonize images acquired on devices with different resolutions. Another application is to convert activity images into images of absorbed dose rate using a radionuclide specific dose kernels with CT density corrections.
3. *Multi-region segmentation* This tool can open most common medical imaging modalities and threshold the images to generate an isosurface of a volume. The resultant isosurface could then depict bone, tumors, organs of interest, etc. By using these isosurfaces as masks, we can derive new boundaries that correspond to a local percent (or SUV) threshold level, e.g., 42% of each individual peak, regardless of peak height and background. The resultant regions can be exported as a mask and/or as RT structures.
4. *3D printing* Isosurfaces and volume renderings generated from images can be exported as.stl files or bitmap (.bmp) images, respectively, for 3D printing. Bitmap files can be saved with user-defined slice thicknesses. This technique allows the user to manipulate and visualize volumes in 3D, prior to performing a 3D print.

### Special features workflow examples

#### Multi-fusion

**Fig. 14:**
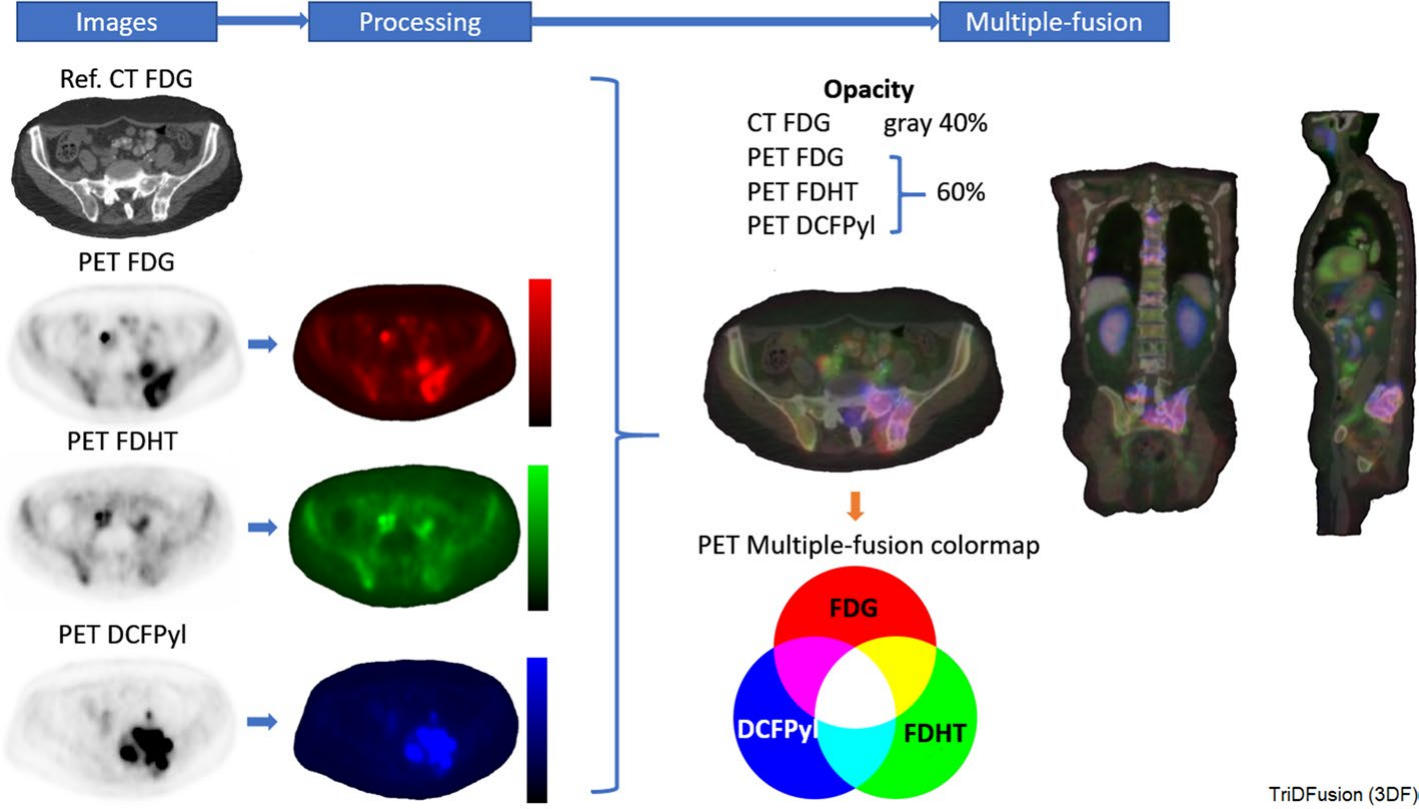
Multi-fusion of three positron emission tomography (PET) images fused with a computed tomography (CT) image of a patient with castrate-resistant metastatic prostate cancer using radiopharmaceuticals: FDHT, FDG, and DCFPyl. FDG, fluorodeoxyglucose; FDHT, fluoro-dihydrotestosterone; and DCFPyl, 18F-piflufolastat

Figure 14 shows the multi-fusion tool in which a reference CT is fused with three PET images. Three different radiotracers that target different biological attributes of prostate cancer are shown: FDG in red for glucose metabolism, FDHT in green for androgen receptors, and DCFPyl in blue for prostate-specific membrane androgen receptors. Each tracer is independently scaled to ensure that its relative importance is displayed. The fusion is performed by sequentially alpha blending each of the three registered PET studies to the reference CT. Using red/green/blue, the three tracers are combined as a RGB colormap. In the final image, a color burst (fully saturated color) is used on the dominant color(s) to visualize regions where tracers overlap as per the Venn diagram in the figure. The thresholds for determining tracer inclusion are user-defined as are the window/level settings. Because all images are fused to the same reference, the activity concentration or SUV from any co-registered pixel (or ROI) can be displayed and saved. Window/leveling and normalization between tracers can be performed on the fly.

#### Convolution using a user-defined 3D kernel

**Fig. 15:**
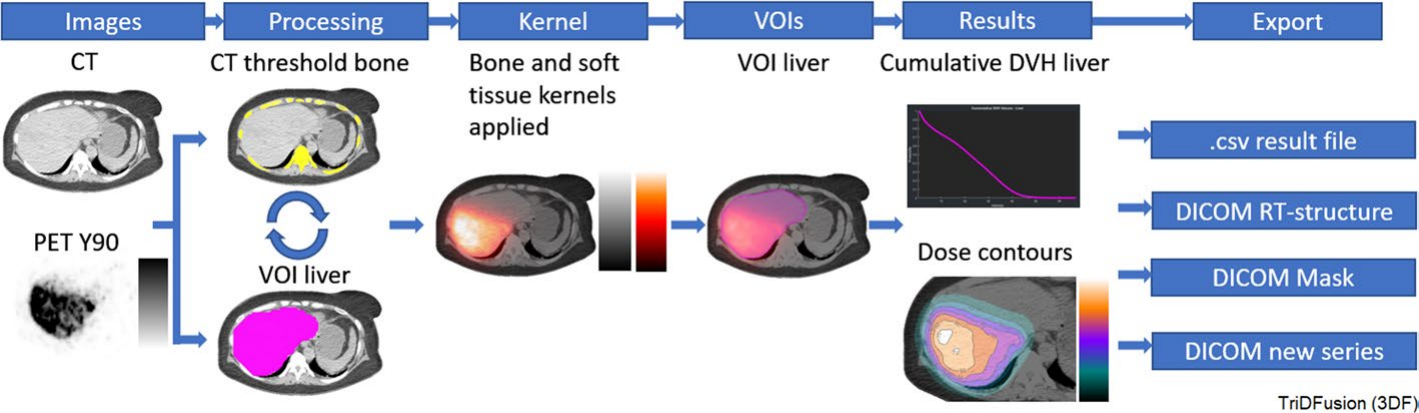
Y90 dosimetry workflow using computed tomography (CT) and positron emission tomography (PET) images. DVH, dose-volume histogram; VOI, volume of interest

Figure 15 shows the $^{90}$Y dosimetry workflow using the convolution kernel panel. Dose rate images are estimated from activity concentration images (e.g., PET, SPECT). Radionuclide and tissue-specific dose kernels are assigned to their appropriate anatomy based on either CT Hounsfield units or user-defined values within a VOI [26]. For tumor and organ-based dosimetry, the VOIs from the reference, or, auxiliary images, can be imported and overlaid on the resultant dose rate images. The VOIs can be exported as RT structures and DICOM masks. Dose-volume histograms can be generated from the VOIs. Both VOIs and DVH curves can be exported as.csv, including the volume in ml, mean, total dose, min, max, etc. The dose rate images can be exported as a new DICOM series.

#### Total tumor segmentation

**Fig. 16:**
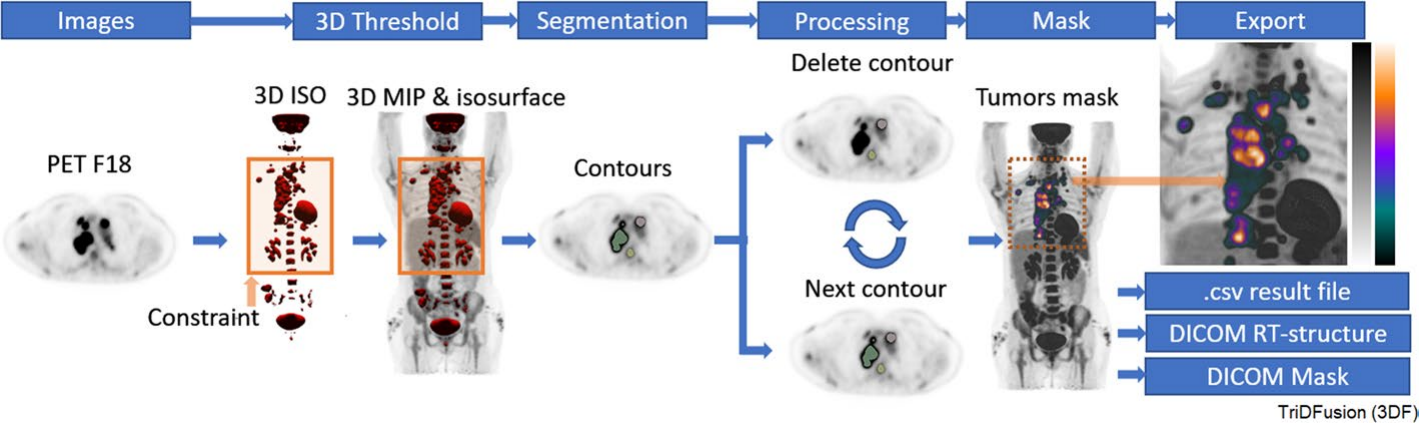
Total tumor segmentation workflow using a positron emission tomography (PET) constrained by a window. MIP, maximum intensity projection. 3D, three-dimensional

The description below follows Fig. 16 from left to right and is an example of the total lesion segmentation workflow. In the first stage, we identify and separate all lesions with elevated radiotracer uptake. This will include organs of high physiologic radiotracer uptake, which can be edited out later. This is accomplished by generating 3D isosurfaces from the PET images. In this example, we use 3D region growing with a 10% threshold (user adjustable) to generate isosurfaces of individual candidate lesions. The resultant isosurfaces are then fused with a MIP of the PET images. This allows physicians and researchers to visualize the candidate lesions and readjust the threshold to ensure capture of all lesions and separation from other structures containing elevated radiotracer uptake if necessary. These isosurface boundaries represent masks where each individual boundary isosurface becomes an independent object. Additional boundary constraints can be applied to avoid large physiological image features such as brain or bladder uptake. In the second step, the user can then independently segment each object based on either a SUV or a percent threshold of each object’s maximum. In this example, the default 42% local threshold was used. When more than one peak exists within an object, a local default threshold of 20% of that object’s maximum peak is used to find additional peaks. These additional peaks are segmented using their local maxima. To be included, VOIs must exceed a minimum default volume of 0.3 ml. In the third stage, the resultant local segmentations are sequentially evaluated (superior to inferior) via the VOI deletion (*Previous*, *Delete*, *Next*) portion of the image segmentation panel (Fig. 2c). Deleted regions are removed from the global mask. The resultant VOIs can be exported as RT structures, DICOM mask, or.csv (total metabolic volume in ml, total lesion glycolysis, total dose, min, max, etc.).

#### 3D printing

**Fig. 17:**
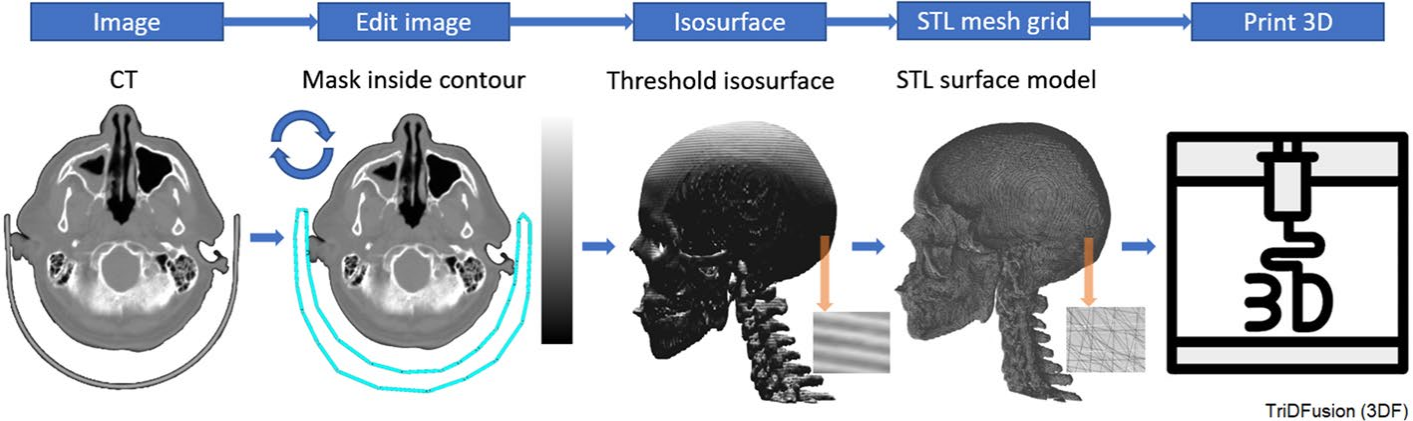
Three-dimensional (3D) printing workflow using a computed tomography (CT) image

Figure 17 shows the generation of 3D print models of a patient’s skull and cervical vertebrae from a CT series. Image editing is performed to remove the couch and head holder by masking within a user-defined VOI. The VOI can be drawn manually or using threshold-based segmentation. A threshold corresponding to certain tissues, such as bone, can be applied to create an isosurface(s). Alternatively, a user-defined segmentation can be used. The resultant isosurface(s) can be exported as.bmp or.stl files suitable for 3D printing and can be visually inspected at any orientation prior to 3D printing within the 3DF viewer.

## Results and discussion

### TriDFusion (3DF) research viewing platform

This work describes a new research viewing platform that includes several nuclear medicine image processing procedures and operations that are not available or conveniently implemented in other software packages. Some examples demonstrating these novel features of the platform were presented to illustrate versatility. The general philosophy behind 3DF is to provide a viewing platform that give users maximal flexibility while avoiding undue complexity. The goal is to allow researchers/scientists to create customized workflows based on the needs of the imaging research project. To accomplish this a highly versatile, unified, inter-operable set of tools is offered that can be combined to support complex research workflows. Below are some general remarks and a summary of some of the tools discussed above, which have been shown to be particularly useful.

#### 3D visualization

The fusion of MIP and isosurface using TriDFusion has been found to facilitate the detection and characterization of lesions on 18F-FDG PET/CT compared with evaluation on the MIP alone. The addition of volume rendering with a customized color and alpha map may make information about the intensity of the uptake in lesions more apparent.

#### Image registration and resampling

Image registration and resampling of multiple series over time is useful to visualize the evolution of disease. The triangulation is also possible while “gating” the different series over time. The intensity of the different series can be automatically set relative to the min-max pixel values of each series or based on the min-max absolute values of all series. The splash view of the coronal, sagittal, axial, or an all plane display layout is configurable and can be used as display over time.

#### Image reorientation

Animal research studies are often scanned orthogonally with respect to the conventional orientation in the gantry; the software allows the visualization of non-axial MIPs.

#### General segmentation

Threshold-based image segmentation within a mask is a useful technique to confine the segmentation to a specific organ or tissue of interest. For example, in Fig. 9, to measure blood volume from CT, the blood within an ROI is segmented from the background tissue. Another example would be the removal of the bed from a CT image. Segmentation within a mask is also an efficient way to measure tumor volume and counts. Measurements of these segmented subregions can be displayed and saved to include the volume and the total, mean, peak, max, min, and standard deviation of the voxels within the tumor volume.

#### Image masking with segmentation

Even in the absence of DICOM tags, TriDFusion 3D volume display can make patients identifiable via their faces or other anatomical attributes even in the absence of DICOM tags. The mask and image segmentation tools allow users to de-identify patients by masking out identifiable features and saving the resultant masked images as described in Table 1.

## Conclusions

This work addresses an important unmet need in the clinical research nuclear medicine community for a vendor-independent, versatile, nuclear medicine imaging research toolbox that can improve workflow, address clinical problems that are currently unsupported by vendors, and allows the use, development, and dissemination of new and innovative research tools. TriDFusion (3DF) source code is available for download from GitHub(https://github.com/DICOMtools/TriDFusion.git). A MATLAB compiled version is also available for download (https://daniellafontaine.com/sdm_downloads/tridfusion-3df-image-viewer/).

### Copyright and collaboration

TriDFusion (3DF) is free open-source software that has been developed and led by Daniel Lafontaine at Memorial Sloan Kettering Cancer Center. The software may be redistributed and/or modified under the terms of the GNU General Public License, as published by the Free Software Foundation, either version 3 of the License, or (at your discretion) any later version. It is for “educational”, “research”, and “not-for-profit” purposes only, which means any noncommercial and nonclinical use. Any contributed computer code may retain the copyright of TriDFusion (3DF) and is responsible for their use of the code.

## Data Availability

TriDFusion (3DF) source code is available for download from GitHub(https://github.com/DICOMtools/TriDFusion.git). A MATLAB compiled version is also available for download (https://daniellafontaine.com/sdm_downloads/tridfusion-3df-image-viewer/).
